# Edoxaban Safety and Effectiveness in Real-Life Patients with Heart Failure and Atrial Fibrillation: EMAYIC Study

**DOI:** 10.3390/jcm14207272

**Published:** 2025-10-15

**Authors:** Rafael Salguero-Bodes, Miriam Padilla Perez, Arturo Andrés Sánchez, Alberto Esteban-Fernández, Martín García López, Manuel Andrés Aparici Feal, José Luis Santos, Hans Paul Gaebelt, Fernando Arribas, on behalf of the EMAYIC Study Investigators

**Affiliations:** 1 Cardiology Department, 12 October University Hospital, 28041 Madrid, Spain; farribasmd@gmail.com; 2 Cardiology Department, Jaén Hospital Complex, 23007 Jaén, Spain; miriam_panarea@yahoo.com; 3 Clinical Cardiology Unit, Ernest Lluch Martin Hospital, 50300 Calatayud, Spain; arturox89@gmail.com; 4 Cardiology Department, Severo Ochoa University Hospital, 28914 Madrid, Spain; athalbertus@gmail.com; 5 Cardiology Department, Doctor José Molina Orosa University Hospital, 35500 Arrecife, Spain; mgl.trasancos@gmail.com; 6 Cardiology Department, Cardiology Clinic Dr. Aparici Feal, 15005 A Coruña, Spain; maparicicardio@hotmail.com; 7 Cardiology Department, Zamora Health Care Complex, 49022 Zamora, Spain; jlsantosiglesias@gmail.com; 8 Cardiology Department, Jiménez Díaz Foundation University Hospital, 28040 Madrid, Spain; hpgaebelt@fjd.es

**Keywords:** edoxaban, heart failure, major or clinically relevant non-major (CRNM) bleeding, atrial fibrillation, Spain

## Abstract

**Background/Objectives:** Real-world data about clinical characteristics and edoxaban performance in patients with heart failure (HF) and atrial fibrillation (AF) are lacking. The EMAYIC study aimed to assess and compare the profile and cardiovascular outcomes in those patients according to HF subtypes based on left ventricular ejection fraction (LVEF). **Methods:** Multicentre, prospective (follow-up: 12 months), observational study. Consecutive adult patients were included at cardiology and internal medicine clinics across Spain with HF (NT-proBNP > 600 pg/mL) and AF, receiving edoxaban as per routine clinical practice. Incidence of major or clinically relevant non-major (CRNM) bleeding and composite of incidence of stroke or systemic embolism (SE) were assessed according to HF subtypes: reduced (HFrEF, LVEF < 40%), mildly reduced (HFmrEF, LVEF40–49%), and preserved (HFpEF, LVEF ≥ 50%) left ventricular ejection fraction. **Results:** Between March 2021 and January 2022, 497 patients were enrolled (HFrEF: 30.4%, HFmrEF: 17.3%, HFpEF: 52.3%). The median age was 76.3 years, 57.9% were male, and the mean CHA2DS2-VASc score was 4. A 60 mg edoxaban dose was prescribed in 70% of patients. The observed rate of bleeding was 6.6% (95% CI: 4.5–9.3%), without differences across HF subtypes (HFrEF: 7.5%, HFmrEF: 3.6%, HFpEF: 7.1%; *p* = 0.474). Intracranial bleeding occurred in one patient (HFrEF). Stroke occurred in seven patients (1.5%) (HFrEF: 3, HFmrEF: 1, HFpEF: 3), two cases of which were fatal (HFrEF: 1, HFpEF: 1). No SE events were reported. Cardiovascular death occurred in 19 patients (4.1%) (HFrEF: 4.8%, HFmrEF: 3.6%, HFpEF: 3.8%; *p* = 0.871). **Conclusions:** This study evidences a low incidence of major or CRNM bleeding in patients with HF and AF treated with edoxaban, regardless of HF subtype. Low rates of stroke (1.5%) and SE events (0%) were assessed.

## 1. Introduction

The coexistence of heart failure (HF) and atrial fibrillation (AF) is associated with poor clinical outcomes [1,2]. HF increases the risk of stroke or systemic embolism (SE) in patients with AF, [3] as reflected in the CHADS_2_ and CHA_2_DS_2_-VASc scores commonly used to assess thromboembolic risk [4]. Moreover, mortality is significantly elevated when HF coexists with AF [5,6]. In those studies, a specific risk is given because the definition of HF may be clinically influenced by a certain degree of heterogeneity, which may cause biases when detecting patients with the disease.

Oral anticoagulation (OAC) remains the cornerstone therapy for preventing thromboembolic events in patients with AF. Despite OAC therapy, the residual risk of cardiovascular events remains high in this population [7]. For decades, vitamin K antagonists (VKA) have been the standard long-term OAC; their use is complicated by a narrow therapeutic range and frequent monitoring requirements [8]. Direct-acting oral anticoagulants (DOAC) have demonstrated comparable efficacy to VKA in prevention of stroke, with an improved safety profile, particularly through a reduced risk of intracranial hemorrhage (ICH) [9,10,11,12,13]. Consequently, current guidelines recommend DOAC over VKA for stroke prevention in patients with AF [4]. This is particularly relevant for patients with HF, who often require concomitant medications that may interfere with VKA metabolism. HF is also an independent risk factor for reduced time within the therapeutic range, thereby limiting the clinical benefits of VKA [14,15].

The use of DOAC to mitigate the risk of stroke or SE in patients with AF and HF has been extensively studied. Secondary analyses from pivotal-phase III DOAC trials [16,17,18,19], as well as pooled analysis of patients with both AF and HF, demonstrated a reduction in major events compared to warfarin, supporting the preferential use of DOAC in this high-risk population [20,21]. However, data regarding real-world use of DOAC in patients with AF and HF remain limited. A small observational study evaluating rivaroxaban reported a low incidence of ischemic or bleeding events [22].

For edoxaban, an oral reversible direct factor Xa inhibitor, a sub-analysis of the Effective Anticoagulation with Factor Xa Next Generation in Atrial Fibrillation–Thrombolysis in Myocardial Infarction 48 (ENGAGE AF-TIMI 48) trial, demonstrated comparable efficacy in stroke/SE prevention and a similar safety profile (in terms of major bleeding) to warfarin in patients with and without HF, regardless of HF severity [19].

A sub-analysis of The Edoxaban Treatment in routiNe clinical prActice for patients with nonvalvular AF in Europe (ETNA-AF-Europe) study [23], a post-authorization observational study evaluating the benefits and risks of edoxaban in European patients with AF, compared outcomes of patients with and without HF, as well as according to left ventricular ejection fraction (LVEF). The 2-year follow-up analysis showed similar rates of ischemic events across groups and HF subtypes (LVEF ≥ 40% or <40%). However, patients with HF exhibited higher rates of major bleeding and cardiovascular and all-cause mortality, with no relevant differences among HF subtypes [23].

When comparing HF prevalence between ENGAGE AF-TIMI 48 and ETNA-AF-Europe, the proportion of patients with HF was higher in the former (58%) than in the latter (14.1%), largely explained by the use of different definitions between studies to form HF cohorts.

Given the limited availability of real-world evidence, this prospective observational study was designed to assess the clinical characteristics, incidence of bleeding and stroke/SE events, and outcomes of patients with AF and “true” HF receiving edoxaban under routine clinical practice in Spain. Moreover, it compared the results across patients according to HF subtypes based on LVEF.

## 2. Materials and Methods

### 2.1. Study Design, Patients, and Endpoints

The EMAYIC study was a multicentre, 12-month follow-up prospective, observational cohort study conducted at cardiology and internal medicine outpatient clinics across Spain.

The study enrolled consecutive adult patients (aged ≥ 18 years) with AF who were treated with edoxaban under routine clinical practice within the preceding three months prior to inclusion and had been diagnosed with HF based on the 2016 European Society of Cardiology (ESC) guidelines for the diagnosis and treatment of acute and chronic HF [24]. According to the data referenced in guidelines, based on cutoff values used in trials [24,25], all enrolled patients were required to have an undoubtedly elevated blood natriuretic peptide concentration (N-terminal pro-brain natriuretic peptide NT-proBNP), arbitrarily set at >600 pg/mL to minimize the risk of false-positive HF diagnosis. Patients were excluded if they had rheumatic moderate or severe mitral valve stenosis, prosthetic heart valves, and/or atrial flutter.

Eligible patients were prospectively followed for 12 months or until early withdrawal for any reason. Baseline data included demographics, cardiovascular risk factors, comorbidities, bleeding history, New York Heart Association (NYHA) functional class [26], AF-related data (e.g., AF type), prior OAC treatment, vital signs, laboratory parameters (blood count, renal and hepatic function, and natriuretic peptides levels), and local cardiac imaging data routinely performed, including LVEF. Information regarding edoxaban therapy, including initial dose, dose modifications, and discontinuation with reasons, was also recorded. Stroke risk was assessed using the CHA2DS2-VASc score [27], and bleeding risk was evaluated with the HAS-BLED score [28].

The primary safety endpoint was the incidence of major or clinically relevant nonmajor (CRNM) bleeding within 12 months, as defined by the International Society on Thrombosis and Haemostasis (ISTH) criteria [29,30,31]. The primary effectiveness endpoint was a composite of stroke or SE within 12 months. Secondary endpoints included clinical outcomes observed during the follow-up period.

The study was conducted in accordance with the World Medical Association Declaration of Helsinki and with national regulations. The study protocol was approved by the Investigation Ethics Committee of 12 de Octubre University Hospital (Madrid) and the corresponding health authorities. Written informed consent was obtained from all participants prior to enrolment.

### 2.2. Statistical Considerations

Quantitative variables were described using measures of central tendency and dispersion, including the mean, standard deviation (SD), median, and interquartile range (IQR). Qualitative variables were presented as counts and percentages. Comparisons of categorical variables were performed using the Chi-square test or Fisher’s exact test, as appropriate.

Safety and efficacy outcomes were assessed for the overall study population and across HF groups. The primary analysis focused on the HF groups as defined in the 2016 guidelines [24]: HF with reduced LVEF (<40%) (HFrEF), HF with midrange or mildly reduced LVEF (40–49%) (HFmrEF), and HF with preserved LVEF (≥50%) (HFpEF). A prespecified analysis combined data from patients with HFmrEF and HFrEF (HFnpEF, HF with non-preserved ejection fraction), comparing this combined group to patients with HFpEF, based on the LVEF threshold of 50% as long as the 2016 guidelines defined that patients with HFmrEF most probably have primarily mild systolic dysfunction, then sharing some pathophysiological characteristics with HFrEF patients.

Missing data were not considered in the analyses, and statistical significance was set at a *p*-value < 0.05. All statistical analyses were performed using the Statistical Package for the Social Sciences (SPSS) version 17.0 (SPSS Inc., Chicago, IL, USA).

## 3. Results

### 3.1. Patients

**Figure 1:**
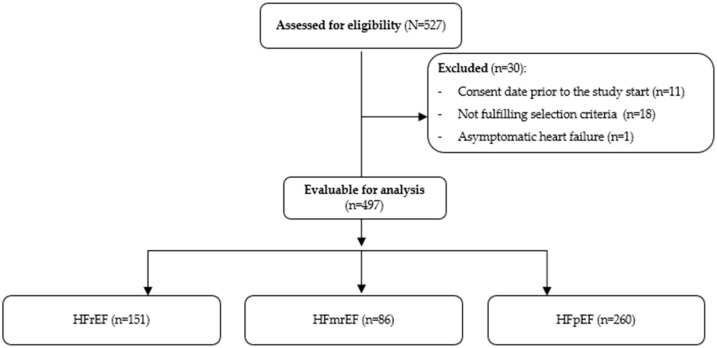
Study patient flow chart. HFmrEF: Heart failure with midrange ejection fraction; HFrEF: Heart failure with reduced ejection fraction; HFpEF: Heart failure with preserved ejection fraction.

Between March 2021 and January 2022, a total of 527 patients were enrolled in the study. Of these, 30 patients were excluded due to non-compliance with eligibility criteria. Consequently, 497 patients were evaluable and included in the final analysis (HFrEF: 151 [30.4%], HFmrEF: 86 [17.3%], HFpEF: 260 [52.3%]) (Figure 1).

The demographic and clinical characteristics of the overall population are summarized in Table 1, while characteristics of HF groups based on LVEF thresholds of 50% (≥50% for HFpEF; <50% for combined HFnpEF) are detailed in Table S1. Compared to patients with HFmrEF and HFpEF, those with HFrEF were younger and predominantly male and had a higher proportion of smokers (active or former) and alcohol users. They also exhibited lower blood pressure, lower CHA_2_DS_2_-VASc scores, and higher NT-pro-BNP levels.

### 3.2. Medical Therapy

Out of the patients who had previously received OAC therapy (52.6%), 81.5% had been treated with VKA with a median time in a therapeutic range (TTR) of 50%, showing no significant differences between HF groups (Table S2).

All patients were receiving edoxaban prior to enrolment, with a median time from diagnosis of AF and HF to edoxaban initiation of 1.4 ± 2.8 years, shorter in HFpEF patients (HFrEF: 1.7, HFmrEF: 1.5, HFpEF: 1.3; *p* = 0.004).

The initial edoxaban dose was 60 mg daily in 70.2% of patients, with no significant differences across HF subgroups (*p* =0.313). One patient received an off-label dose of edoxaban (90 mg) (Table S3). Among patients meeting the recommended criteria for dose adjustment, 85.4% received a 30 mg dose.

### 3.3. Follow up

A total of 378 patients (87.1%) completed the study’s 12-month follow-up. Early withdrawal occurred in 56 patients (12.9%), with similar proportions across HF groups, mainly due to death (HFrEF: 12 [63.2%], HFmrEF: 5 [41.7%], HFpEF: 16 [64.0%]) (Figure 1). No patients withdrew consent, nor were any discontinued due to safety concerns.

### 3.4. Safety

Major or CRNM bleeding was reported in 31 patients (6.6%; 95% CI: 4.5–9.3%), with no significant differences across HF subgroups (HFrEF: 11 [7.5%], HFmrEF: 3 [3.6%], HFpEF: 17 [7.1%]; *p* = 0.474). A post hoc multivariate analysis identified anemia as the only factor associated with major or CRNM bleeding (Table S4). Gastrointestinal bleeding was the most frequent event, accounting for 58.1% of cases (Table 2). Similarly, no significant differences were observed when combining patients with HFrEF and HFmrEF (LVEF < 50%) and comparing them to HFpEF (Table S5).

Overall, nine patients (1.8%) experienced 10 treatment-related adverse events (AEs) (HFrEF: n = 2, HFrEF: n = 0, HFpEF: n = 8). None of these AEs were fatal (Table 3).

### 3.5. Effectiveness

Overall, seven patients (1.5%) experienced a stroke (ischemic: n = 3; transient ischemic attack (TIA): n = 3; haemorrhagic: n = 1), with a similar incidence across HF groups (HFrEF: n = 3 [2.0%], HFmrEF: n = 1 [1.2%], HFpEF: n = 3 [1.3%]). Stroke was fatal in two cases (HFrEF: n = 1, HFpEF: n = 1). No SE events were reported during the follow-up period (Table 2).

Combined analysis of HFrEF and HFmrEF versus HFpEF also showed no significant differences (Table S5). Post hoc Kaplan–Meier survival curves for stroke and major or CRNM bleeding are shown in Figures S1 and S2.

### 3.6. Other Outcomes

The overall mortality rate during the 12-month follow-up was 6.6% (33/497).

Group analysis showed similar death rates across HF types HFrEF (12/151, 7.9%), HFmrEF (5/86, 5.8%), and HFpEF (16/260, 6.2%). Cardiovascular mortality specifically was also similar among groups (HFrEF: 7 [4.8%], HFmrEF: 3 [3.6%], HFpEF: 9 [3.8%]; *p* = 0.871) (Table 2).

Overall, 28.6% (n = 142) of patients required hospitalization during follow-up, primarily due to HF exacerbation (Table S6).

Additionally, a total of 54 cardiovascular surgical or interventional procedures had been performed during the follow-up period (Table S6).

## 4. Discussion

Although AF and HF frequently coexist, leading to a higher risk of cardiovascular events and increased mortality rates, real-world studies designed to investigate patients with both conditions remain limited [19,22,23].

To our knowledge, this is the first study specifically designed to assess major or CRNM bleeding and thromboembolic outcomes in patients with HF and AF treated with edoxaban in a real-world setting. A primary analysis was conducted based on HFrEF, HFmrEF, and HFpEF groups, defined ad hoc at the time of study design in accordance with the 2016 ESC guidelines [4].

Additionally, a post hoc analysis was performed using an LVEF threshold of 50%, combining HFrEF and HFmrEF into one group and comparing it to HFpEF. This approach aligns with evidence suggesting that HFmrEF patients may benefit from similar therapies as those with HFrEF [32,33].

The main findings of this study suggest that the risk of hemorrhagic and embolic events in HF and AF patients treated with edoxaban is relatively low, which is in agreement with the literature [19,23,32,33]. Moreover, in our study, despite baseline differences among HF subgroups, there were no significant differences in ischemic/SE events, bleeding or hospitalization rates due to HF, or cardiovascular mortality.

These results are consistent with data from randomized controlled trials (RCTs) and real-world studies [19,23,32,33]. The ENGAGE AF-TIMI 48 trial [19] was a randomized, double-blind, double-dummy study comparing two once-daily regimens of edoxaban with warfarin in 21,105 patients diagnosed with moderate- to high-risk AF. A subanalysis of ENGAGE categorized HF based on electronic case report forms completed by local investigators, adhering to the American College of Cardiology (ACC) and American Heart Association (AHA) definitions [34]. Participants were divided into three groups: those without HF, those with mild HF (NYHA classes I–II), and those with severe HF (NYHA classes III–IV) [19]. Based on these criteria, 58% of the patients were classified as having HF, with 45% in NYHA classes I–II and 13% in classes III–IV. Among these patients, 49.5% had an LVEF below 50%. LVEF data were available for 68% of patients without HF and 79% of those with HF [19]. HF was associated with increased adjusted hazard ratios for stroke, SE, major and fatal bleeding, all-cause and cardiovascular mortality, and hospitalizations. However, the relative efficacy and safety of edoxaban compared with well-managed warfarin were consistent between patients with and without HF [19]. Edoxaban was associated with lower rates of major bleeding, fatal bleeding, and net clinical outcomes (death, stroke/SE, or major bleeding) although gastrointestinal bleeding was slightly higher than with warfarin.

The ETNA-AF-Europe study provided valuable insights into the real-world application of edoxaban [23]. This multicentre, prospective, observational study enrolled 13,980 patients over a follow-up period of four years, aiming to evaluate the safety of edoxaban by monitoring bleeding events and assessing efficacy through the recording of major adverse cardiovascular events. Patients with documented structural or functional cardiac abnormalities were classified as having HF, which included those with congestive HF, ischemic cardiomyopathy, LVEF below 40%, or frequent dyspnoea without chronic obstructive pulmonary disease (COPD) [23]. HF patients were further subdivided by LVEF: <40% or ≥40%. Over the two-year follow-up, ischemic event rates were similar between patients with and without HF [23]. However, HF patients had a higher incidence of major bleeding, cardiovascular mortality, and overall mortality. Interestingly, no significant differences were observed in ischemic or bleeding events between the HF subtypes, although mortality was notably higher in patients with LVEF < 40% [23].

One major limitation of the abovementioned studies [19,23] is the use of different and non-standardized criteria for defining HF, which lacked systematic assessment of relevant information such as LVEF. This led to different population characteristics and a significantly varied proportion of HF patients (58% vs. 14.1%), making direct comparisons between groups challenging. These inconsistencies hinder the ability to draw robust conclusions about safety, efficacy, and clinical outcomes, especially in the real-life setting.

When comparing the results with those of ETNA-AF-Europe (the only study with available comparable data) [23], the overall percentage of patients experiencing major or CRNM bleeding was low in EMAYIC (6.6%), albeit higher than among the 1854 HF patients in ETNA-AF-Europe (3.0%). Regarding the LVEF subgroups, this difference was more pronounced in HFrEF patients (7.5% in EMAYIC versus 2.59% among 671 patients with LVEF < 40% in ETNA-AF-Europe). In the HFmrEF and HFpEF subgroups, EMAYIC reported rates of 3.6% and 7.1%, respectively, compared to 3.17% among 857 patients with LVEF ≥ 40% in ETNA-AF-Europe. The overall stroke rate in EMAYIC was 1.5%, compared to 0.86% in ETNA-AF-Europe (a result combining any stroke or SE) [23]. Subgroup analysis showed a higher stroke rate in HFrEF patients (2.0% in EMAYIC versus 0.51% in ETNA-AF-Europe). Rates in HFpEF patients were 1.2% and 1.3%, respectively, compared to 0.93% in ETNA-AF-Europe patients with LVEF ≥ 40% [23]. CV mortality rates were comparable between EMAYIC (4.1%) and ETNA-AF-Europe (4.87%). Among HFrEF patients, CV mortality was slightly lower in EMAYIC (4.8%) than in ETNA-AF-Europe (5.99%) [23]. Similarly, HFmrEF and HFpEF patients in EMAYIC had lower CV mortality rates (3.6% and 3.8%, respectively) compared to patients with LVEF ≥ 40% in ETNA-AF-Europe (4.14%).

These differences may be explained by the fact that EMAYIC included a large, representative cohort of real-world patients with “true” HF, subgrouped according to current guidelines and stringent inclusion criteria to minimize false positives and enhance specificity in HF diagnosis (i.e., NT-proBNP ≥ 600 pg/mL). Because of that reason, patients included in EMAYIC may represent a different population, exhibiting bleeding and embolic risks slightly different from those described in previous studies with edoxaban, strengthening the importance of conducting real-life studies reflecting true clinical settings.

However, NT-proBNP levels are influenced by age, renal function, and BMI, complicating their interpretation, particularly in older populations [35,36]. For instance, prior research suggests that older individuals may naturally have elevated NT-proBNP levels even in the absence of HF [35]. Furthermore, NT-proBNP levels can be significantly elevated in patients with AF, even in the absence of HF [36].

Most patients received an appropriate edoxaban dose from the beginning of the study, reflecting good adherence to clinical recommendations in real-world practice [4].

It is necessary to note that in our study, a trend toward higher bleeding and stroke incidence in patients with HFrEF was observed. Potential mechanisms that may explain this finding include altered platelet function, the presence of left ventricular thrombus, or pharmacokinetic variability in this subgroup.

In addition, our study identified anemia as a factor independently associated with major or CRNM bleeding. This finding is in agreement with found in previous studies [37,38].

Real-world studies evaluating the use of DOACs (other than edoxaban) in patients with AF and HF are also limited [22,39]. For instance, the FARAONIC study, aimed at determining AEs and mortality risk factors in 672 patients with AF and HF treated with rivaroxaban in Spain, revealed a low incidence of thromboembolic events (2.9%), acute coronary syndromes (2.0%), major bleeding (3.1%), and intracranial bleeding but no fatalities (0.5%) [22]. Moreover, the Italian registry in the setting of atrial fibrillation ablation with rivaroxaban (IRIS), with data from 250 consecutive AF patients eligible for catheter ablation on rivaroxaban, showed no major bleeding during the 12-month follow-up [39].

On the other hand, although HFmrEF might intuitively be considered an intermediate phenotype between HFrEF and HFpEF, available evidence indicates a more complex and variable profile [40]. Early studies (such as OPTIMIZE-HF [41] and ADHERE [42]) suggested that HFmrEF resembled HFpEF in terms of age, hypertension, atrial fibrillation, and comorbidities, with intermediate patterns for sex and ischemic etiology. However, later investigations (SwedeHF Registry [43] and the CHARM program [44]) demonstrated greater similarity to HFrEF, especially regarding younger age, male sex, ischemic etiology, kidney disease, diabetes, and prior myocardial infarction. In addition, the pathophysiology of HFmrEF remains unknown [45]. Current evidence suggests that HFmrEF may arise from progressive worsening of left ventricular function in HFpEF patients, particularly in those with concomitant coronary artery disease, which contributes to declining LVEF. Alternatively, HFmrEF can result from a recovery of the systolic function in patients with HFrEF [45]. The strengths of the EMAYIC study include its multicentric, prospective observational design. This allowed direct comparisons of cardiovascular outcomes, including stroke, bleeding, and mortality, over a 12-month period in a large cohort of real-world patients with HF and AF. However, prescribing edoxaban under clinical practice conditions resulted in an uneven distribution of patients across subgroups, reflecting real-world variability.

Study limitations: Patients included in this study were representative of the HF and AF population in Spain, and results may not be generalizable to other countries and clinical settings, for example, those with atrial flutter. Moreover, the exclusive reliance on NT-proBNP levels for HF diagnosis in recruited patients resulted in enhanced specificity; however, it also might limit generalizability by excluding patients with clinical HF but lower biomarker levels, particularly in HFpEF, where natriuretic peptides can be paradoxically lower. Another limitation derives from the follow-up duration. While the 12-month follow-up adequately captures short-term safety signals, the clinical relevance would be strengthened by longer-term data on main endpoints, such as mortality and stroke incidence, particularly given the chronic nature of both AF and HF. Additionally, the study was not powered to detect differences in clinical outcomes in the post hoc analysis using an LVEF threshold of 50% (HFrEF or HFmrEF) versus HFpEF, though no evidence of significant differences was observed. The observational design and effort to avoid interfering with routine care limited the systematic collection of laboratory and imaging data, which should be considered when interpreting these findings.

## 5. Conclusions

This real-world study demonstrates a comparable incidence of major or CRNM bleeding, stroke, SE events, and CV death among patients with HF and AF treated with edoxaban, irrespective of LVEF.

## Figures and Tables

**Table 1:** Patient’s sociodemographic and clinical characteristics in the overall population and in HF groups.

Characteristics	Overall	HFrEF	HFmrEF	HFpEF	*p*-Value
N (%)	497 (100.0)	151 (30.4)	86 (17.3)	260 (52.3)	
Age, median (IQR), years ^a^	76.3 (67.6–82.5)	71.0 (63.2–78.3)	77.2 (69.0–81.4)	78.4 (70.8–84.9)	<0.001 *
Gender, male, n (%) ^b^	288 (57.9)	122 (80.8)	51 (59.3)	115 (44.2)	<0.001 ^Ω^
BMI, median (IQR), Kg/m^2^	28.7 (25.2–31.1) ^a^	28.0 (25.4–31.2)	28.1 (25.8–30.4) ^b^	27.7 (24.8–31.2) ^c^	0.867 *
Smoking, n (%)					<0.001 ^Ω^
Never smoker	295 (59.4)	63 (41.7)	52 (60.5)	180 (69.2)	
Ex-smoker	152 (30.6)	65 (43.0)	27 (31.4)	60 (23.1)	
Active smoker	50 (10.1)	23 (15.2)	7 (8.1)	20 (7.7)	
Alcohol consumption, n (%) ^d^	30 (6.0)	16 (10.6)	3 (3.5)	11 (4.2)	0.018 ^Ω^
Comorbidities, n (%) ^e^					
Arterial hypertension	371 (77.5) ^f^	110 (73.3) ^g^	62 (74.7) ^h^	199 (80.9) ^i^	
Congestive HF	361 (75.4) ^f^	130 (86.7) ^g^	64 (77.1) ^h^	167 (67.9) ^i^	
Dyslipidemia	266 (55.5) ^f^	89 (59.3) ^g^	45 (54.2) ^h^	132 (53.7) ^i^	
CKD (eGFR < 60 mL/min)	172 (35.9) ^f^	56 (37.3) ^g^	27 (32.5) ^h^	89 (36.2) ^i^	
Diabetes mellitus	163 (34.0) ^f^	57 (38.0) ^g^	30 (36.1) ^h^	76 (30.9) ^i^	
Ischemic cardiomyopathy	101 (21.1) ^f^	54 (36.0) ^g^	19 (22.9) ^h^	28 (11.4) ^i^	
Myocardial infarction	70 (69.3)	42 (77.8)	11 (57.9)	17 (60.7)	0.138 ^Ω^
CAD	29 (77.5) ^f^	52 (34.7) ^g^	19 (22.9) ^h^	28 (11.4) ^i^	
Valvular heart disease	94 (18.9)	24 (15.9)	15 (17.4)	55 (21.2)	0.393 ^Ω^
Mitral	49 (52.1)	14 (58.3)	7 (46.7)	28 (50.9)	
Aortic	37 (39.4)	9 (37.5)	7 (46.7)	21 (38.2)	
Prior ischemic stroke	20 (4.2) ^f^	7 (4.7) ^g^	3 (3.6)	10 (4.1) ^i^	0.636 ^†^
Prior TIA	4 (18.2)	0 (0.0)	1 (33.3)	3 (25.0)	
Anemia	47 (9.5)	9 (6.0)	13 (15.1)	25 (9.6)	0.068 ^Ω^
Labile INR (TTR < 60%)	133 (27.8) ^f^	47 (31.3) ^g^	21 (25.3) ^h^	65 (26.4) ^i^	
Bleeding predisposition	28 (5.6)	7 (4.6)	6 (7.0)	15 (5.8)	0.747 ^Ω^
Bleeding history (in previous year)	17 (3.4)	3 (2.0)	5 (5.8)	9 (3.5)	0.296 ^Ω^
Major bleeding	7 (41.2)	1 (33.3)	2 (40.0)	4 (44.4)	0.564 ^†^
Moderate-severe dementia	5 (1.0)	2 (1.3)	1 (1.2)	2 (0.8)	0.848 ^†^
Clinical characteristics					
Blood pressure, median (IQR), mmHg					
SBP	126.0 (115.0–137.0)	119.0 (108.0–129.0)	129.5 (117.8–140.0)	130.0 (120.0–140.0)	<0.001 *
DPB	75.0 (69.0–82.0)	72.0 (64.8–80.0)	76.0 (70.0–84.3)	77.0 (70.0–83.0)	0.002 *
Type of NVAF, n (%)					0.496 ^Ω^
Paroxysmal	126 (25.4)	38 (25.2)	23 (26.7)	65 (25.0)	
Persistent	125 (25.2)	40 (26.5)	25 (29.1)	60 (23.1)	
Long-standing persistent	28 (5.6)	12 (7.9)	5 (5.8)	11 (4.2)	
Permanent	218 (43.9)	61 (40.4)	33 (38.4)	124 (47.7)	
CHAD_2_DS_2_-VASc score, mean (SD)	4.0 (1.5)	3.8 (1.6)	4.1 (1.5)	4.1 (1.5)	0.213 *
HAS-BLED score, mean (SD)	1.5 (0.9) ^j^	1.5 (1.0) ^k^	1.6 (1.0)	1.6 (0.9)	0.579 *
HF LVEF **(%)**, median (IQR)	50.0 (35.0–60.0)	30.0 (25.0–35.0)	45.0 (42.0–45.0)	60.0 (55.0–62.0)	<0.001 *
NYHA Functional Classification, n (%)					0.602 ^†^
Class I	107 (22.9)	35 (23.8)	24 (28.9)	48 (20.2)	
Class II	291 (62.2)	92 (62.6)	49 (59.0)	150 (63.0)	
Class III	63 (13.5)	17 (11.6)	10 (12.0)	36 (15.1)	
Class IV	7 (1.5)	3 (2.0)	0 (0.0)	4 (1.7)	
Laboratory					
Creatinine clearance, median (IQR), mL/min	64.0 (49.0–80.0) ^l^	62.0 (48.0–79.0) ^m^	66.0 (52.0–80.0)	65.0 (48.0–81.0) ^n^	0.659 ^€^
Cockroft-Gault, median (IQR), mL/min/m^2^	60.8 (45.4–82.9)	64.9 (47.8–93.1)	62.4 (49.3–86.5)	57.7 (43.4–78.8)	0.052
CKD-EPI, median (IQR), mL/min/1.73 m^2^	64.1 (47.3–79.9)	61.5 (46.7–76.7)	67.3 (51.5–80.9)	63.8 (46.3–80.4)	0.269 *
NT-pro-BNP, median (IQR)	1884.0 (1009.0–3371.5)	2272.5 (1178.3–4078.8)	1750.0 (934.0–3480.5)	1708–0 (994.3–2857.0)	0.005 *

HF: heart failure; HFrEF, heart failure with reduced ejection fraction; HFmrEF, heart failure with mid-range ejection fraction; HFpEF, heart failure with preserved ejection fraction; IQR, interquartile range; BMI, body mass index; CKD, chronic kidney disease; eGFR, estimated glomerular filtration rate; INR, international normalized ratio; TTR, time in therapeutic range; CAD, coronary artery disease; TIA; transient ischemic attack; CRNMB, clinically relevant nonmajor bleeding; NVAF; non-valvular atrial fibrillation; HF FEVI Heart Failure With Preserved Ejection Fraction; CKD-EPI, Chronic Kidney Disease Epidemiology Collaboration; SBP, systolic blood pressure; DPB, diastolic blood pressure. N available: ^a^ 492, ^b^ 84, ^c^ 257, ^f^ 479, ^g^ 150, ^h^ 83, ^i^ 246, ^j^ 496, ^k^ 150, ^l^ 490, ^m^ 147, ^n^ 257. ^d^ More than 8 alcoholic drinks per week; ^e^ More frequent comorbidities (>10% of patients). Statistical procedures: * Kruskal–Wallis test; ^Ω^ Chi-square test; ^†^ Fisher Exact test; ^€^ ANOVA.

**Table 2:** Safety and efficacy outcomes in the overall population in HF groups.

Characteristics	Overall	HFrEF	HFmrEF	HFpEF	*p*-Value
Bleeding outcomes					
Major or CRNM bleeding, n %	31 (6.6)	11 (7.5)	3 (3.6)	17 (7.1)	0.474
Major bleeding					
Number of bleeding events, n (%)	11 (2.4) ^a^	5 (3.4) ^b^	1 (1.2) ^c^	5 (2.1) ^d^	0.602 ^†^
1	10 (90.9)	5 (100.0)	1 (100.0)	4 (80.0)	
2	1 (9.1)	0 (0.0)	0 (0.0)	1 (20.0)	
Median number if events (IQR)	1.0 (1.0–1.0)	1.0 (1.0–1.0)	1.0 (1.0–1.0)	1.0 (1.0–1.5)	0.549 *
Type of events, n (%)					
Gastrointestinal	8 (72.7)	3 (60.0)	1 (100.0)	4 (80.0)	
Intracranial	1 (9.1)	1 (20.0)	0 (0.0)	0 (0.0)	
Other	2 (18.2)	1 (20.0)	0 (0.0)	1 (20.0)	
CRNM bleeding	21 (4.5) ^a^	6 (4.1) ^b^	3 (3.6) ^c^	12 (5.0) ^d^	0.829 ^Ω^
Number of bleeding events, n (%)					0.353 ^†^
1	16 (76.2)	6 (100.0)	2 (66.7)	8 (66.7)	
2	3 (14.3)	0 (0.0)	0 (0.0)	3 (25.0)	
3	2 (9.5)	0 (0.0)	1 (33.3)	1 (8.3)	
Median number of events (IQR)	1.0 (1.0–1.5)	1.0 (1.0–1.0)	1.0 (1.0–X)	1.0 (1.0–2.0)	0.287 *
Type of events, n (%)					
Gastrointestinal		3 (50.0)		7 (58.3)	
Epistaxis		0 (0.0)	2 (66.7)	1 (8.3)	
Hematuria		1 (16.7)	0 (0.0)	2 (16.7)	
Other		2 (33.3)	0 (0.0)	0 (0.0)	
Minor bleeding					
Number of bleeding events, n (%)	26 (5.6) ^a^	4 (2.7) ^b^	5 (6.0) ^c^	17 (7.1) ^d^	0.180 ^Ω^
1	21 (80.8)	4 (100.0)	3 (60.0)	14 (82.4)	
2	3 (11.5)	0 (0.0)	1 (20.0)	2 (11.8)	
3–4	2 (7.7)	0 (0.0)	1 (20.0)	1 (5.9)	
Median number of events (IQR)	1.0 (1.0–1.0)	1.0 (1.0–1.0)	1.0 (1.0–1.0)	1.0 (1.0–1.0)	0.291 *
Type of events, n (%)					
Gastrointestinal		0 (0.0)	2 (40.0)	4 (23.5)	
Epistaxis		1 (25.0)	0 (0.0)	2 (11.8)	
Hematuria		1 (25.0)	3 (60.0)	1 (5.9)	
Other		2 (50.0)	2 (40.0)	7 (41.2)	
Cardiovascular death					
Death due to CV causes, n (%)	19 (4.1)	7 (4.8)	3 (3.6)	9 (3.8)	0.871 ^Ω^
Reason, n (%)					
HF		3 (42.9)	2 (66.7)	7 (77.8)	
Stroke		1 (14.3)	0 (0.0)	0 (0.0)	
ICH		1 (14.3)	0 (0.0)	0 (0.0)	
Other		2 (28.6)	1 (33.3)	2 (22.2)	
Thromboembolic events					
Stroke, n (%)	7 (1.5) ^a^	3 (2.0) ^b^	1 (1.2) ^c^	3 (1.3) ^d^	0.876 ^†^
Stroke event, n (%)					0.486 ^†^
Ischemic stroke	3 (42.9)	2 (66.7)	1 (100.0)	0 (0.0)	
Hemorrhagic stroke	1 (14.3)	0 (0.0)	0 (0.0)	1 (33.3)	
TIA	3 (42.9)	1 (33.3)	0 (0.0)	2 (66.7)	
Stroke outcome, n (%)					
Disabling	2 (28.6)	1 (33.3)	0 (0.0)	1 (33.3)	>0.999 ^†^
Fatal	2 (28.6)	1 (33.3)	0 (0.0)	1 (33.3)	>0.999 ^†^
Systemic embolism, n (%)	0 (0.0)	0 (0.0)	0 (0.0)	0 (0.0)	-

HFrEF, heart failure with reduced ejection fraction; HFmrEF, heart failure with mid-range ejection fraction; HFpEF, heart failure with preserved ejection fraction; TIA, transient ischemic attack; IQR, interquartile range; CRNMB, clinically relevant nonmajor bleeding; HF, heart failure. N available: ^a^ 468, ^b^ 147, ^c^ 83, ^d^ 238. Statistical procedures: * Kruskal–Wallis test; ^Ω^ Chi-square test; ^†^ Fisher Exact test.

**Table 3:** Adverse events other than bleeding in the overall population and in HF groups.

**Characteristics**	**Overall**	**HFrEF**	**HFmrEF**	**HFpEF**	***p*** **-Value**
Total number of adverse events	10	2	0	8	
Treatment-related adverse events, n (%)	9 (1.8)	2 (1.3)	0 (0.0)	7 (2.7)	
Adverse events, n (%) ^a^					
Anemia	6 (1.2)	0 (0.0)	0 (0.0)	5 (1.9)	
Diarrhea	2 (0.4)	1 (0.7)	0 (0.0)	0 (0.0)	
Exanthema	1 (0.2)	0 (0.0)	0 (0.0)	1 (0.4)	
Acute kidney injury secondary to diarrhea	1 (0.2)	1 (0.7)	0 (0.0)	0 (0.0)	
Abdominal pain	1 (0.2)	0 (0.0)	0 (0.0)	1 (0.4)	

HFrEF, heart failure with reduced ejection fraction; HFmrEF, heart failure with mid-range ejection fraction; HFpEF, heart failure with preserved ejection fraction; CRNMB, clinically relevant nonmajor bleeding. ^a^ One patient could experience more than one adverse event.

## Data Availability

The data presented in this study are available on request from the corresponding author.

## Supplementary Materials

The following supporting information can be downloaded at https://www.mdpi.com/article/10.3390/jcm14207272/s1. Table S1: Patient’s sociodemographic and clinical characteristics in the overall population and in HF groups (LVEF threshold of 50%); Table S2: Prior anticoagulant treatment in the overall population and in HF groups; Table S3: Treatment with edoxaban in the overall population and in HF groups; Table S4: Results of the bivariate and multivariate analysis of factors associated with major or CRNM bleeding. Table S5: Safety and efficacy outcomes in the overall population in HF groups (LVEF threshold of 50%); Table S6: Hospitalization and cardiovascular procedures in the overall population and in HF groups.. Figure S1; Kaplan-Meier survival curve for stroke; Figure S2: Kaplan-Meier survival curve for major or CRNM bleeding.

## Abbreviations

The following abbreviations are used in this manuscript:

ACC	American College of Cardiology
ACC	American Heart Association
AEs	Adverse events
AF	Atrial fibrillation
BMI	Body mass index
CAD	Coronary artery disease
CKD	Chronic kidney disease
CKD-EPI	Chronic Kidney Disease Epidemiology Collaboration
CRNM	Clinically relevant nonmajor
CRNMB	Clinically relevant nonmajor bleeding
DOAC	Direct-acting oral anticoagulants
DPB	Diastolic blood pressure
eGFR	Estimated glomerular filtration rate
ESC	European Society of Cardiology
HF	Heart failure
HF FEVI	Heart Failure With Preserved Ejection Fraction
HFmrEF	Heart failure with mid-range ejection fraction
HFpEF	Heart failure with preserved ejection fraction
HFrEF	Heart failure with reduced ejection fraction
ICH	Intracranial hemorrhage
INR	International normalized ratio
IQR	Interquartile range
ISTH	International Society on Thrombosis and Haemostasis
LVEF	Left ventricular ejection fraction
NVAF	Non-valvular atrial fibrillation
NYHA	New York Heart Association
OAC	Oral anticoagulation
SBP	Systolic blood pressure
SD	Standard deviation
SE	Systemic embolism
TIA	Transient ischemic attack
TTR	Therapeutic range
VKA	Vitamin K antagonists
