# Supplementary material for: Edoxaban Safety and Effectiveness in Real-Life Patients with Heart Failure and Atrial Fibrillation: EMAYIC Study
Source: J Clin Med. 2025 Oct 15;14(20):7272. doi: 10.3390/jcm14207272 (PMC12565050; doi:10.3390/jcm14207272)
Supplement: Supplementary file 1 [file jcm-14-07272-s001.zip › jcm-3830726-supplementary.pdf]

SUPPLEMENTARY TABLES

Supplementary Table S1. Patient´s sociodemographic and clinical characteristics in the overall population and in HF groups (LVEF threshold of 50%)

**Characteristics**	**Overall**	**HFnpEF**	**HFpEF**	**p-value**
No. (%)	497 (100.0)	237 (47.7)	260 (52.3)	
**Age**, median (IQR), years	76.3 (67.6-82.5)	74.3 (64.5-79.5)	78.4 (70.8-84.9)	<0.001 *
**Gender**, male, n (%)	288 (57.9)	173 (73.0)	115 (44.2)	<0.001 Ω
**BMI**, median (IQR), Kg/m^2	28.7 (25.2-31.1)	28.0 (25.5-30.8)	27.7 (24.8-31.2)	0.597 *
**Smoking**, n (%)				<0.001 Ω
Never smoker	295 (59.4)	115 (48.5)	180 (69.2)	
Ex-smoker	152 (30.6)	92 (38.8)	60 (23.1)	
Active smoker	50 (10.1)	30 (12.7)	20 (7.7)	
**Alcohol consumption**, n (%) [a]	30 (6.0)	19 (8.0)	11 (4.2)	0.077 Ω
**Comorbidities**, n (%) [b]				
Arterial hypertension	371 (77.5)	172 (73.8)	199 (80.9)	

Congestive HF	361 (75.4)	194 (83.3)	167 (67.9)	
Dyslipidemia	266 (55.5)	134 (57.5)	132 (53.7)	
CKD (eGFR <60 ml/min)	172 (35.9)	83 (35.6)	89 (36.2)	
Diabetes mellitus	163 (34.0)	87 (37.3)	76 (30.9)	
Ischemic cardiomyopathy	101 (21.1)	73 (31.3)	28 (11.4)	
Myocardial infarction	70 (69.3)	53 (72.6)	17 (60.7)	0.246 Ω
CAD	42 (41.6)	29 (39.7)	13 (46.4)	0.541 Ω
Valvular heart disease	94 (18.9)	39 (16.5)	55 (21.2)	0.182 Ω
Mitral	49 (52.1)	21 (53.8)	28 (50.9)	
Aortic	37 (39.4)	16 (41)	21 (38.2)	
Ischemic stroke	20 (90.9)	10 (100)	10 (83.3)	0.481 †
TIA	4 (18.2)	1 (10.0)	3 (25.0)	
Anemia	47 (9.5)	22 (9.3)	25 (9.6)	0.899 Ω
Labile INR (TTR <60%)	133 (27.8)	68 (29.2)	65 (26.4)	

Bleeding predisposition	28 (5.6)	13 (5.5)	15 (5.8)	0.891 Ω
Bleeding history (in last year)	17 (3.4)	8 (3.4)	9 (3.5)	0.958 Ω
Major bleeding	7 (41.2)	3 (37.5)	4 (44.4)	0.440 †
Moderate-severe dementia	5 (1.0)	3 (1.3)	2 (0.8)	0.673 †
**Clinical characteristics**				
**Blood pressure**, median (IQR), mmHg				
SBP	126.0 (115.0-137.0)	121.0 (110.0-134.0)	130.0 (120.0-140.0)	<0.001 *
DBP	75.0 (69.0-82.0)	74.0 (65.3-81.8)	77.0 (80.0-73.0)	
**Type of NVAF**, n (%)				0.196 Ω
Paroxysmal	126 (25.4)	61 (25.7)	65 (25.0)	
Persistent	125 (25.2)	65 (27.4)	60 (23.1)	
Long-standing persistent	28 (5.6)	17 (7.2)	11 (4.2)	
Permanent	218 (43.9)	94 (39.7)	124 (47.7)	
**CHAD2DS2-VASc score**, mean (SD)	4.0 (1.5)	3.9 (1.6)	4.1 (1.5)	0.277 *

**HAS-BLED score**, mean (SD)	1.5 (0.9)	1.5 (1.0)	1.8 (0.9)	0.612 *
**HF FEVI (%)**, median (IQR)	50.0 (35.0-60.0)	35.0 (30.0-42.0)	60.0 (55.0-62.0)	<0.001 *
**NYHA Functional Classification**, n (%)				0.105 †
Class I	66 (13.3)	24 (10.1)	42 (16.2)	
Class II	305 (61.4)	144 (60.8)	161 (61.9)	
Class III	121 (24.3)	66 (27.8)	55 (21.2)	
Class IV	5 (1.0)	3 (1.3)	2 (0.8)	
**Laboratory**				
Creatinine clearance, median (IQR), mL/min	64.0 (49.0-80.0)	64.0 (50.0-79.0)	65.0 (48.0-81.0)	0.466 €
Creatinine, median (IQR), mg/dL	1.0 (0.8-1.3)	1.1 (0.9-1.4)	1.0 (0.8-1.3)	0.003 *
CKD-EPI, median (IQR), mL/min/1.73 m^2	64.1 (47.3-79.9)	64.3 (49-79.4)	63.8 (46.3-80.4)	0.665 *
NT-pro-BNP, median (IQR)	1884.0 (1009.0-3371.5)	2062.0 (1074.0-3870.5)	1708.0 (994.3-2857.0)	0.011 *

HF: heart failure; HFnpEF, heart failure with non-preserved ejection fraction (HF group combining patients with HFrEF and HFmrEF (LVEF<50%); HFpEF: heart failure with preserved ejection fraction; IQR, interquartile range; BMI, body mass index; CKD, chronic kidney disease; eGFR, estimated glomerular filtration rate;

INR, international normalized ratio; TTR, time in therapeutic range; CAD, coronary artery disease; TIA; transient ischemic attack; CRNMB, clinically relevant nonmajor bleeding; NVAF; non-valvular atrial fibrillation; HF FEVI Heart Failure With Preserved Ejection Fraction; CKD-EPI, Chronic Kidney Disease Epidemiology Collaboration; SBP, systolic blood pressure; DPB, diastolic blood pressure.

[a] More than 8 alcoholic drinks per week; [b] More frequent comorbidities (>10% of patients).

Statistical procedures: [*] Mann-Whitney, [Ω] Chi-square test, [†] Fisher Exact test, [€] t-test.

Supplementary Table S2. Prior anticoagulant treatment in the overall population and in HF groups

Characteristics	Overall	HFrEF	HFmrEF	HFpEF	p-value
No. (%)	497 (100.0)	151 (30.4)	86 (17.3)	260 (52.3)	
**Prior OAC**, n (%)	260 (52.6) [a]	86 (57.0)	42 (49.4) [b]	132 (51.2) [c]	0.426 [Ω]
**Type of OAC**, n (%)					0.558 [Ω]
DOAC	48 (18.5)	18 (20.9)	9 (21.4)	21 (15.9)	
VKA	212 (81.5)	68 (79.1)	33 (78.6)	111 (84.1)	
**DOAC therapy**					
Type, n (%)					0.014 [†]
Rivaroxaban	18 (37.5)	12 (66.7)	3 (33.3)	3 (14.3)	
Apixaban	23 (47.9)	5 (27.8)	5 (55.6)	13 (61.9)	
Dabigatran	7 (14.6)	1 (5.6)	1 (11.1)	5 (23.8)	
Treatment duration, median (IQR), months	9.7 (3.7-34,8)	19.8 (4.7-37.7)	8.9 (4.3-23.4)	4.5 (1.5-23.9)	0.310 [*]

**VKA therapy**					
Type, n (%)					0.729 †
Acenocoumarol	203 (95.8)	65 (95.6)	31 (93.9)	107 (96.4)	
Warfarin	9 (4.2)	3 (4.4)	2 (6.1)	4 (3.6)	
TTR, mean (SD), %	48.4 (17.1)	44.7 (15.3)	46.8 (18.2)	51.5 (17.6)	0.197 *
Treatment duration, median (IQR), months	38.0 (7.8-91,0)	38.4 (7.0-79.8)	26.5 (7.7-81.0)	38.8 (8.1-95.2)	0.841 *

DOAC, direct oral anticoagulant; HFrEF, heart failure with reduced ejection fraction; HFmrEF, heart failure with mid-range ejection fraction; HFpEF, heart failure with preserved ejection fraction; IQR, interquartile range; OAC, oral anticoagulant; SD, standard deviation; TTR: time to therapeutic range; VKA, vitamin K antagonist.

N available: [a] 494, [b] 85, [c] 258. Statistical procedures: * Kruskal Wallis test; Ω Chi-square test; † Fisher Exact test.

Supplementary Table S3. Treatment with edoxaban in the overall population and in HF groups

Characteristics	Overall	HFrEF	HFmrEF	HFpEF	p-value
No. (%)	497 (100.0)	151 (30.4)	86 (17.3)	260 (52.3)	
**Time since diagnosis of AF and HF**, median (IQR), yr	*1.4 (2.8)* [a]	*1.7 (2.9)* [b]	*1.5 (3.3)* [c]	*1.3 (2.7)* [d]	*0.004* [*]
**Dose at treatment initiation**, n (%), mg					0.313 [†]
30	147 (29.6)	39 (25.8)	22 (25.6)	86 (33.1)	
60	349 (70.2)	112 (74.2)	64 (74.4)	173 (66.5)	
90	1 (0.2)	0 (0.0)	0 (0.0)	1 (0.4)	
Median (IQR), mg	60.0 (30.0-60.0)	60.0 (30.0-60.0)	60.0 (30.0-60.0)	60.0 (30.0-60.0)	0.230 [*]
**Criteria for dose reduction**, n (%)					
At least one criterion					
Weight ≤60 Kg	40 (8.1) [e]	7 (4.6)	6 (7.0)	27 (10.4) [f]	0.170 [Ω]
CrCl 15-50 ml/min	132 (26.6)	43 (28.5)	18 (20.9)	71 (27.3)	0.416 [Ω]
Concomitant use of cyclosporine, dronedarone, erythromycin, and/or ketoconazole	1 (0.2)	0 (0.0)	0 (0.0)	1 (0.4)	>0.999 [†]

**Reduced dose**, n (%), mg	157 (31.7) [e]	46 (30.5)	23 (26.7)	88 (34.9) [f]	0.427 [Ω]
30	134 (85.4)	37 (80.4)	20 (87.0)	77 (87.5)	
60	23 (14.6)	9 (19.6)	3 (13.0)	11 (12.5)	
Median (IQR), mg	30.0 (30.0-30.0)	30.0 (30.0-30.0)	30.0 (30.0-30.0)	30.0 (30.0-30.0)	0.534 [*]
**Treatment modification**, n (%)	24 (5.7)	9 (7.1)	6 (8.0)	9 (4.1)	0.335 [Ω]
No. of changes, n (%)					0.707 [†]
1	22 (91.7)	7 (77.8)	6 (100.0)	9 (100.0)	
2	1 (4.2)	1 (11.1)	0 (0.0)	0 (0.0)	
3	1 (4.2)	1 (11.1)	0 (0.0)	0 (0.0)	
Median no, of changes, (IQR)	1.0 (1.0-1.0)	1.0 (1.0-1.5)	1.0 (1.0-1.0)	1.0 (1.0-1.0)	0.176 [*]
**Treatment duration,** median (IQR), months	5.5 (2.0-9.0)	5.2 (2.7-9.3)	6.6 (1.2-7.9)	5.4 (1.3-9.1)	0.818 [*]
**Treatment discontinuation**, n (%)	49 (9.9)	20 (13.2)	9 (10.5)	20 (7.7)	-
**Subsequent treatment**, n (%)	14 (28.6)	5 (25.0)	3 (33.3)	6 (30.0)	
Type, n (%)					

Apixaban	8 (57.1)	3 (60.0)	2 (66.7)	3 (50.0)	>0.999
Rivaroxaban	2 (14.3)	1 (20.0)	0 (0.0)	1 (16.7)	
VKA (acenocoumarol)	4 (28.6)	1 (20.0)	1 (33.3)	2 (33,3)	
Reason, n (%)					
Cardiovascular issues or bleeding events	5 (35.7)	1 (20.0)	1 (33.3)	3 (50.0)	

HFrEF, heart failure with reduced ejection fraction; HFmrEF, heart failure with mid-range ejection fraction; HFpEF, heart failure with preserved ejection fraction; AF, atrial fibrillation; HF, heart failure; IQR, interquartile range; CrCl, creatinine clearance; VKA: vitamin K antagonists.

Statistical procedures: * Kruskal Wallis test; Ω Chi-square test; † Fisher Exact test.

Supplementary Table S4. Results of the bivariate and multivariate analysis of factors associated with major or CRNM bleeding

	**B**	**SE**	**p-value**	**HR**	**HR IC95% inferior**	**HR IC95% superior**
**Independent variables in bivariate analysis**						
Concomitant medication potentially predisposing to bleeding at the start of edoxaban treatment? (0=No, 1=Yes)	-0.702	0.734	0.339	0.496	0.118	2.090
Use of antiplatelet agents at baseline (0=No, 1=Yes)	-0.276	0.734	0.707	0.759	0.180	3.198
Prior oral anticoagulation before starting edoxaban (0=No, 1=Yes)	0.222	0.382	0.562	1.248	0.590	2.641
Liver disease (0=No, 1=Yes)	0.686	0.734	0.350	1.986	0.471	8.371
Diabetes mellitus (0=No, 1=Yes)	0.126	0.394	0.750	1.134	0.523	2.457
Age	0.000	0.017	0.991	1.000	0.967	1.035
Hypertension (0=No, 1=Yes)	0.474	0.494	0.337	1.607	0.611	4.229
Kidney disease (Glomerular filtration rate < 60 mL/min) (0=No, 1=Yes)	0.698	0.378	**0.065**	2.010	0.957	4.221

Stroke (0=No, 1=Yes)	0.551	0.734	0.453	1.735	0.412	7.313
Anemia (0=No, 1=Yes)	1.366	0.419	**0.001**	3.920	1.725	8.912
History of bleeding (previous major or clinically relevant non-major bleeding) (0=No, 1=Yes)	0.880	1.019	0.388	2.410	0.327	17.769
Low body weight <50 kg (0=No, 1=Yes)	-3.024	6.969	0.664	0.049	0.000	41610.460
Alcohol consumption (≥ 8 alcoholic drinks per week) (0=No, 1=Yes)	0.809	0.611	**0.186**	2.245	0.677	7.442
Labile INR (time in therapeutic range < 60%) (0=No, 1=Yes)	0.393	0.394	0.319	1.482	0.684	3.211
**Multivariate analysis**						
Anemia (0=No, 1=Yes)	1.366	0.419	**0.001**	3.920	1.725	8.912

Supplementary Table S5. Safety and efficacy outcomes in the overall population in HF groups (LVEF threshold of 50%)

Characteristics	Overall	HFnpEF	HFpEF	p-value
**Bleeding outcomes**				
**Major or CRNM bleeding, %**				
**Major bleeding**				
Number of bleeding events, n (%)	11 (2.4)	6 (2.6)	5 (2.1)	0.717 Ω
1	10 (90.9)	6 (100.0)	4 (80.0)	
2	1 (9.1)	0 (0.0)	1 (20.0)	
Median number if events (IQR)	1.0 (1.0-1.0)	1.0 (1.0-1.0)	1.0 (1.0-1.5)	0.455 †
Type of events, n (%)				
Gastrointestinal	8 (72.7)	4 (66.7)	4 (80.0)	
Intracranial	1 (9.1)	1 (16.7)	0 (0.0)	
Other	2 (18.2)	1 (16.7)	1 (20.0)	
**CRNM bleeding**	21 (4.5)	9 (3.9)	12 (5.0)	0.555 Ω

Number of bleeding events, n (%)				0.355 †
1	16 (76.2)	8 (88.9)	8 (66.7)	
2	3 (14.3)	0 (0.0)	3 (25.0)	
3	2 (9.5)	1 (11.1)	1 (8.3)	
Median number of events (IQR)	1.0 (1.0-1.5)	1.0 (1.0-1.0)	1.0 (1.0-2.0)	0.317 *
Type of events, n (%)				
Gastrointestinal		3 (33.3)	7 (58.3)	
Epistaxis		2 (22.2)	1 (8.3)	
Hematuria		1 (11.1)	2 (16.7)	
Other		2 (22.2)	0 (0.0)	
**Minor bleeding**				
Number of bleeding events, n (%)	26 (5.6)	9 (3.9)	17 (82.4)	0.127 Ω
1	21 (80.8)	7 (77.8)	14 (60.0)	
2	3 (11.5)	1 (11.1)	2 (11.8)	

3-4	2 (7.7)	1 (11.1)	0 (0.0)	
Median number of events (IQR)	1.0 (1.0-1.0)	1.0 (1.0-1.5)	1.0 (1.0-1.0)	0.724 [*]
Type of events, n (%)				
Gastrointestinal		2 (22.2)	4 (23.5)	
Epistaxis		1 (11.1)	2 (11.8)	
Hematuria		4 (44.4)	1 (5.9)	
Other		2 (22.2)	6 (35.3)	
**Thromboembolic events**				
**Stroke**, n (%)	7 (1.5)	4 (1.7)	3 (1.3)	0.720 [†]
Stroke event, n (%)				0.229 [†]
Ischemic stroke	3 (42.9)	3 (75.0)	0 (0.0)	
Hemorrhagic stroke	1 (14.3)	0 (0.0)	1 (33.3)	
TIA	3 (42.9)	1 (25.0)	2 (66.7)	
Stroke outcome, n (%)				

Disabling	2 (28.6)	1 (25.0)	1 (33.3)	>0.999 †
Fatal	2 (28.6)	1 (25.0)	1 (33.3)	>0.999 †
**Systemic embolism**, n (%)	0 (0.0)	0 (0.0)	0 (0.0)	-
**Cardiovascular death**				
Death due to CV causes, n (%)	19 (4.1)	10 (4.3)	9 (3.8)	0.756 Ω
Reason, n (%)				
HF		5 (50.0)	6 (66.7)	
Stroke		1 (10.0)	0 (0.0)	
ICH		1 (10.0)	0 (0.0)	
Other		3 (30.0)	3 (33.3)	

HFrEF, heart failure with reduced ejection fraction; HFmrEF, heart failure with mid-range ejection fraction; HFpEF, heart failure with preserved ejection fraction; TIA, transient ischemic attack; IQR, interquartile range; CRNMB, clinically relevant nonmajor bleeding; HF, heart failure; ICH, intracranial hemorrhage.

Statistical procedures: * Mann-Whitney, Ω Chi-square test, † Fisher Exact test.

Supplementary Table S6. Hospitalization and cardiovascular procedures in the overall population and in HF groups

Characteristics	Overall	HFrEF	HFmrEF	HFpEF	p-value
**Hospitalization**, n (%)	142 (30.3) [a]	54 (36.7) [b]	23 (27.7) [c]	65 (27.3) [d]	0.308 †
Reason, n (%)					
HF	38 (26.8)	14 (25.9)	5 (21.7)	19 (29.2)	*ND*
CAD	3 (2.1)	1 (1.9)	1 (4.3)	1 (1.5)	
PAD	4 (2.8)	2 (3.7)	0 (0.0)	2 (3.1)	
Ischemic stroke	2 (1.4)	1 (1.9)	1 (4.3)	0 (0.0)	
TIA	2 (1.4)	0 (0.0)	0 (0.0)	2 (3.1)	
CRNMB	11 (7.7)	4 (7.4)	2 (8.7)	5 (7.7)	
Major bleeding	4 (2.8)	1 (1.9)	1 (4.3)	2 (3.1)	
Intracranial hemorrhage	2 (1.4)	1 (1.9)	0 (0.0)	1 (1.5)	
**Cardiovascular procedures**, n (%)		28 (62.2)	9 (40.9)	17 (44.7)	
Type of cardiovascular procedures, n (%)					

Heart rate			
AF ablation	6 (21.4)	1 (11.1)	1 (5.9)
Ablation of other substrates	0 (0.0)	0 (0.0)	1 (5.9)
Cardioversion	1 (3.6)	2 (22.2)	1 (5.9)
Rhythm device	9 (32.1)	1 (11.1)	3 (17.6)
Coronary procedures			
Coronariography	4 (14-3)	3 (33.3)	2 (11.8)
Coronary intervention	3 (10.7)	0 (0.0)	0 (0.0)
Non-coronary interventional procedures			
Structural interventionism	3 (10.7)	1 (11.1)	6 (35.3)
Peripheral interventionism	1 (11.1)	1 (11.1)	2 (11.8)
Transplant	1 (11.1)	0 (0,0)	1 (5.9)

HFrEF, heart failure with reduced ejection fraction; HFmrEF, heart failure with mid-range ejection fraction; HFpEF, heart failure with preserved ejection fraction; TIA, transient ischemic attack; IQR, interquartile range; CRNMB, clinically relevant nonmajor bleeding; HF, heart failure; CAD, coronary arterial disease; PAD, peripheral arterial disease.

N available: [a] 468, [b] 147, [c] 83, [d] 238. Statistical procedures: * Kruskal Wallis test; Ω Chi-square test; † Fisher Exact test.

Supplementary Figure S1. Kaplan-Meier survival curve for stroke

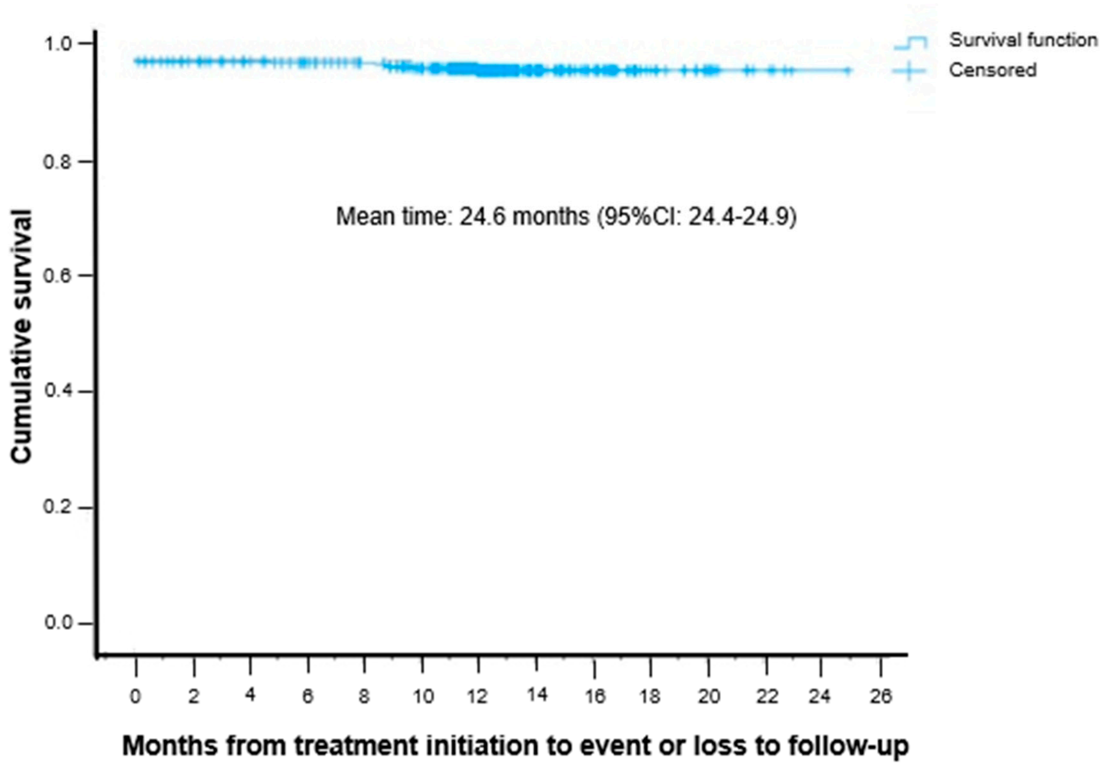

Supplementary Figure S2. Kaplan-Meier survival curve for major or CRNM bleeding

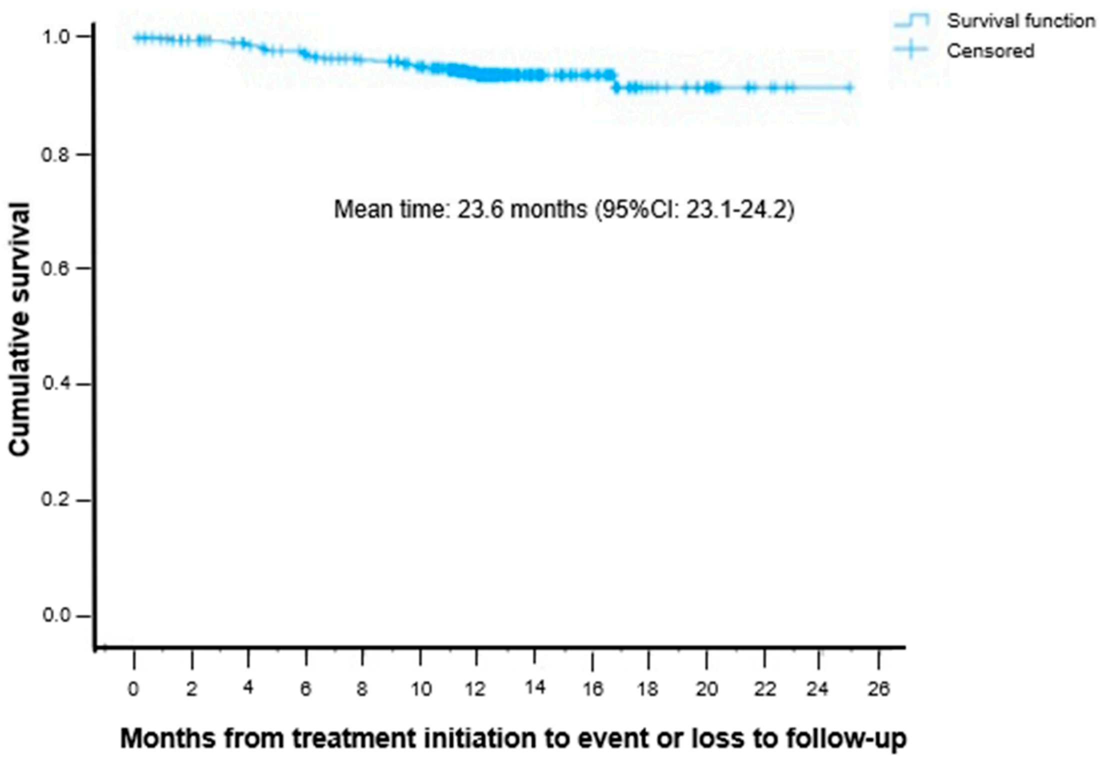

CRNM: Clinically relevant nonmajor
